# Evaluation of microRNAs as liquid biopsy markers in adrenocortical tumors

**DOI:** 10.3389/fendo.2025.1511520

**Published:** 2025-01-30

**Authors:** Chrysoula Mytareli, Vassiliki Kalotychou, Alexandros Lafioniatis, Gregory Kaltsas, George N. Zografos, Athina Markou, Labrini Papanastasiou, Athanasios Fountas, Dimitra Argyro Vasilliadi, Evanthia Kassi, Marina Mantzourani, Anna Angelousi

**Affiliations:** ^1^ First Department of Internal Medicine, European Reference Network on Rare Endocrine Conditions (ENDO-ERN), Laikon General Hospital, Medical School, National and Kapodistrian University of Athens, Athens, Greece; ^2^ Department of Propaedeutic and Internal Medicine, European Reference Network on Rare Endocrine Conditions (ENDO-ERN), Laikon General Hospital, National and Kapodistrian University of Athens, Athens, Greece; ^3^ First Department of Surgery, ‘G. Gennimatas’ General Hospital of Athens, Athens, Greece; ^4^ Unit of Endocrinology, and Diabetes Center, ‘G. Gennimatas’ General Hospital of Athens, Athens, Greece; ^5^ Department of Endocrinology, Diabetes and Metabolism, European Reference Network on Rare Endocrine Conditions (ENDO-ERN), Evangelismos Hospital, Athens, Greece; ^6^ Department of Biological Chemistry, Medical School, National and Kapodistrian University of Athens, Athens, Greece

**Keywords:** adrenocortical tumors, diagnosis, follow-up, molecular biomarker, liquid biopsy, micro-RNAs

## Abstract

**Introduction:**

Adrenal tumors (ATs) encompass a wide differential diagnosis, necessitating a multi-step process for accurate identification. Liquid biopsy emerges as a promising non-invasive technique for distinguishing between malignant and benign, as well as hyperfunctioning and non-functioning cases. Recent studies have highlighted the potential of microRNAs as circulating biomarkers; however, their clinical utility remains underexplored. This study aims to validate the diagnostic performance of selected circulating microRNAs, (miR-483-5p, miR-210, miR-335 and miR-22-3p), identified through microRNA profiling studies, as markers of malignancy or cortisol hypersecretion in a cohort of patients with ATs.

**Methods:**

We collected serum samples from 75 patients with ATs, including 50 cases of adrenocortical adenomas (ACA) and 25 cases of adrenocortical carcinomas (ACC), along with 15 controls. In the ACC subgroup, 16 samples were obtained preoperatively or upon detection of recurrence (active ACC group), while the remaining from disease-free patients with long-term follow-up. Among the 56 patients with ATs evaluated preoperatively (50 with ACAs and 6 with ACC), 26 had non-functioning tumors, 22 exhibited mild autonomous cortisol secretion, and 8 had Cushing syndrome. Quantitative real-time polymerase chain reaction was employed to analyze microRNA expression.

**Results:**

Circulating levels of miR-483-5p and miR-210 were significantly elevated in patients with active ACC compared to both ACAs (p<0.001 and p=0.004, respectively) and controls (p=0.02 and p = 0.03, respectively). Notably, miR-483-5p serum levels were higher in patients with active ACC compared to disease-free ACC patients (p = 0.01). MiR-483-5p demonstrated the best diagnostic accuracy for distinguishing active ACC cases from ACAs, achieving a sensitivity of 81.3% and a specificity of 88%, and from disease-free ACC patients, reaching sensitivity of 81.3% and specificity of 89%. MiR-22-3p serum levels successfully differentiated patients with Cushing syndrome from those with non-functioning ATs (area under the curve=AUC=0.800, 95% CI: 0.653–0.953, p=0.01) and controls (AUC= 0.800, 95% CI: 0.610–0.990, p=0.02). Additionally, circulating miR-22-3p levels exhibited a significant correlation with traditional diagnostic tests for hypercortisolism.

**Conclusion:**

This study supports the potential of a liquid biopsy approach as an innovative method for diagnosing and monitoring patients with ATs, offering a complementary tool to existing diagnostic methods.

## 1 Introduction

Adrenal tumors (ATs) are commonly encountered masses with a broad differential diagnosis, including both benign or malignant cases, as well as hormone-secreting or non-functioning tumors. Accurately ruling out malignant or functioning cases is often complex and requires a comprehensive evaluation, including clinical, hormonal, radiological, and histopathological assessment (1).

Adrenocortical carcinoma (ACC) is a rare but aggressive tumor, representing up to 4% of ATs (1). The overall prognosis of ACC is poor and recurrence rates remain high even after complete tumor resection (2). Guidelines recommend post-treatment surveillance involving clinical and hormonal assessment, along with regular radiological imaging for over 5 years (2). Hormonal evaluation is, also, essential in all ATs considering the complications associated with hormone hypersecretion (1). However, reaching a definitive diagnosis is challenging due to the limitations of traditional diagnostic tests.

In the era of precision medicine, liquid biopsy has provided novel insights in the identification of biomarkers for accurate diagnosis and improved monitoring of ATs. Circulating tumor cells and tumor-derived material reflect the molecular profile of corresponding tumor tissue, offering a non-invasive alternative to traditional biopsy (3). Among these biomarkers, micro-RNAs (miRNAs) have shown significant promise. These small non-coding RNAs, primarily involved in the post-transcriptional regulation of gene expression, are found altered in various pathologies, due to chromosomal abnormalities, epigenetic modifications or defects in the miRNA biogenesis machinery (4). Although most miRNAs are found intratumorally, they can be found in body fluids, originating either from the passive release of dead or apoptotic cells or from active secretion, possibly serving as part of intercellular communication (4). Despite being extracellular, miRNAs remain highly stable, making them reliable and accessible as circulating biomarkers (4).

Initial tissue studies using microarray and next-generation sequencing platforms identified distinct miRNA expression profiles across different types of ATs (5). Building on these tissue-based findings, researchers have explored these miRNA profiles in blood samples showing potential for use as non-invasive biomarkers in liquid biopsy (6–13). Nonetheless, these findings have been validated in only a limited number of patients and independent studies using the gold-standard method, quantitative real-time polymerase chain reaction (qRT-PCR), and have yet to be confirmed for clinical application.

The aim of our study is therefore to evaluate the diagnostic performance of selected circulating miRNAs in detecting malignancy and cortisol hypersecretion in a real-world clinical setting of individuals with ATs.

## 2 Materials and methods

### 2.1 Participants

A total of 75 patients with ATs and 15 controls were enrolled in the study from September 2022 to September 2024. This project received approval from the Internal Ethical Review Committee of the Laikon General Hospital (protocol number 4, 17.01.2022) and Gennimatas General Hospital of Athens (protocol number 30519, 21.11.2023). All patients/controls provided written informed consent prior to inclusion. All procedures were in accordance with the Declaration of Helsinki (1964) and its later amendments.

Epidemiological, clinical and biochemical data were retrospectively recorded from the patients’ medical records. All included patients underwent laboratory evaluation and imaging studies, based on the ATs diagnosis and treatment guidelines (1). Fifty adrenocortical adenomas (ACA) were defined according to their imaging and hormonal characteristics or histopathological analysis when available, while all ACC cases were confirmed with histopathologic analysis and were followed according to the recommended protocol outlined in the relevant guidelines (2). Twenty-five ACC patients were further classified into 2 groups based on their disease status at the time of sample collection: those with active disease, either newly diagnosed or with newly detected recurrence (n=16), and those who remained disease-free after long-term follow-up (n=9). Preoperative AT cases (n=56 as a total, n=50 ACA and n=6 ACC) were also subclassified according to functionality into non-functioning, mild autonomous cortisol secretion (MACS) and Cushing syndrome (CS) cases, in line with the recent consensus statement and clinical practice guidelines (1). Specifically, patients with CS were classified based on at least two positive results out of the three recommended tests, along with the clinical phenotype. In contrast, patients with MACS were defined as those with a free serum cortisol value greater than 1.8 µg/dL after the 1-mg overnight dexamethasone suppression test (ODST) and who lacked symptoms of CS (1, 14). Patients diagnosed with other types of ATs (pheochromocytomas, aldosterone-producing adenomas, myelolipomas, cysts, schwannomas, ganglioneuromas, metastases) were excluded from the study, as were those with extraadrenal active malignancies. Patients with normal adrenal glands, based on computed tomography (CT) and/or magnetic resonance imaging (MRI) within the past six months, comprised the control group (n=15). These individuals were followed in the first department of Internal Medicine of Laikon General Hospital for unrelated conditions.

The selection of miRNAs for analysis was guided by a thorough review of the existing literature, which identified miR-483-5p, miR-210, and miR-335 as having the highest diagnostic value for distinguishing ACC from ACAs in both tissue and blood samples (5–8, 10–13), and miR-22-3p for identifying cortisol hypersecretion (9).

### 2.2 Hormone blood measurements

Serum levels of dehydroepiandrosterone sulfate, androstenedione, 17-hydroxyprogesterone, estradiol, cortisol following a 1 mg ODST and 24-hour urinary free cortisol (UFC) were measured using high-performance liquid chromatography-tandem mass spectrometry (HPLC-MS/MS) on a SCIEX Triple Quad 5500+ QTRAP Ready system. Plasma metanephrines were also measured when needed using LC-MS for excluding patients with pheochromocytoma. Additionally, adrenocorticotropic hormone (ACTH) levels as well as plasma aldosterone and renin activity were assessed, when needed, using a chemiluminescent assay (Liaison, DiaSorin).

### 2.3 Experimental methods

Whole blood was collected from patients/controls in a primary blood collection tube without anticoagulants during the morning between 8 AM and 9 AM. After collection and centrifugation, serum was stored at −80 °C until analysis. MiRNAs were isolated from 200 μL serum with miRNeasy serum/plasma advanced kit (Qiagen, Germany) according to the manufacturer’s protocol. In the final step, total cell-free miRNA was eluted in 20 μl RNase-free water and stored at –80°C until further analysis. Afterwards, single-stranded cDNA was synthesized from total miRNA with miRCURY LNA Reverse Trancription Kit (Qiagen, Germany) according to the manufacturer’s protocol. The non-human exogenous cel-miR-39-3p was added to the samples before reverse transcription and used as reference gene for data normalization. Q RT- PCR was performed in triplicate using the diluted (1:5) reverse-transcription product combined with miRCURY LNA miRNA PCR Assays (has-miR-483-5p, has-miR-210, hsa-miR-335, hsa-miR-22-3p) and the miRCURY LNA SYBR^®^ Green PCR Kit. Reactions were amplified on the C1000 Thermal cycler (CFX96 Real Time system, BIO-RAD). Data were analyzed with CFX Manager Software version 3.1 (Bio-Rad). The expression was calculated according to the dCT method [-dCT values=-(CT of target miRNA-CT of internal control miRNA)] (15).

### 2.4 Statistical analysis

The statistical analysis was performed using SPSS version 28.0. In the descriptive analysis, categorical variables were expressed as their absolute and relative frequency. Numerical variables were presented as mean ± SD (Standard Deviation) for normally distributed data, otherwise by median and range. T-test or Mann-Whitney U test was used to compare the continuous variables between two groups, based on the results of Shapiro-Wilk normality test. For the comparison of numerical variables among multiple groups, one-way ANOVA or Kruskal- Wallis test followed by *post-hoc* analysis were used, as appropriate. The chi -square test or Fisher’s exact test was applied to compare categorical variables. Correlations between quantitative variables were investigated using either Pearson’s (r) or Spearman correlation coefficient (r_s_). For each miRNA, receiver operator characteristics (ROC) curves were constructed by plotting the true positive rate (sensitivity) against the false positive rate (1-specificity), and the Area Under Curve (AUC) was calculated to evaluate their diagnostic performance. P-values below 0.05 were considered statistically significant.

## 3 Results

### 3.1 Clinical and epidemiological characteristics of the studied populations

Among the entire cohort of 25 ACC patients, 10 (40%) were male, with a median age of 54.5 years. Preoperative routine hormonal work-up showed that 36% had glucocorticoid excess, 32% had concomitant cortisol and androgen hypersecretion, and the remaining patients had non-functioning tumors. At presentation, 7 ACC cases (28%) were classified as stage II, 13 (52%) as stage III, and 5 (20%) as stage IV, with a median tumor size of 110 mm. All patients underwent adrenalectomy, and histopathological analysis revealed a median Weiss score of 6 and a Ki67 index of 25%. At the time of inclusion, blood samples were collected preoperatively from 6 patients with a final diagnosis of ACC and from 10 patients at recurrence (defined as ‘‘active ACC’’ subgroup), as well as from 9 disease-free patients after a median follow-up period of 55 months (defined as ‘‘disease-free ACC’’ subgroup). In the cohort of 50 ACA patients, 15 (30%) patients were male, with a median age at diagnosis of 64 years. Of these, 25 (50%) had non-functioning ACAs, 3 had CS, and 22 had MACS. Adrenalectomy was performed in 4 patients with either CS or suspicious imaging characteristics. The median tumor size in this group was 25 mm. Among the control group, 4 participants (26.7%) were male, with a median age of 60 years. There were no significant differences in age or sex distribution among the groups. Data from all 4 studied groups are summarized in **Table 1**.

**Table 1:** Baseline epidemiological and clinical characteristics of the studied groups (Patients with active ACC, Disease-free ACC patients, patients with ACA, controls).

Characteristics	Patients with active ACC (n=16)	Disease-free ACC patients (n=9)	Patients with ACA (n=50)	Controls (n=15)	p-value (pairwise comparisons)
Age, years median (range)	54.5 (33–89)	55 (41–72)	64 (25–81)	60 (40–79)	ns
Sex: M, n (%)	6 (24%)	4 (44.4%)	15 (30%)	4 (26.7%)	ns
Initial hormonal status, n (%)	2 (12.5%) nf, 6 (37.5%) C, 8 (50%) C and A	6 (66.7%) nf, 3 (33.3%) C	25 (50%) nf, 25 (50%) C (3 with CS and 22 with MACS)	na	ns
Primary tumor size, mm median (range)	110 (52–185)	105 (58–120)	25 (10–68)	na	<0.001 for the comparison between active ACC and ACA, as well as disease-free ACC and ACA
Follow-up period, months median (range)	12 (12–60)	55 (32–183)	42 (25-80)	na	ns

ACC, adrenocortical carcinoma; ACA, adrenocortical adenoma; MACS, mild autonomous cortisol secretion; CS, Cushing syndrome; nf, non-functioning; C, cortisol-producing; A, androgen-producing; na, not applicable; ns, non significant.

ANOVA, Kruskal-Wallis, chi-square, or Fisher’s exact tests were applied to assess differences between groups as appropriate, followed by *post-hoc* analyses for pairwise comparisons.

The clinical characteristics of the three subgroups of ACC patients, stratified by disease status, are depicted in **Table 2**. In the preoperative ACC group (n=6), most tumors were cortisol-producing (83.4%) and there was a high incidence of advanced-stage disease (50% in stage III and 50% in stage IV). The recurrent ACC group (n=10) predominantly consisted of patients with combined androgen and cortisol-producing tumors (80%). The median initial tumor size was 110 mm, the median Ki67 index was 39%, and the median Weiss score was 6. Additionally, 70% of patients in this group had undergone complete surgical resection (R0). Despite treatment, recurrence occurred after a median of 4 months, with local recurrence observed in 40% of patients and distant metastases in the lungs (20%), liver (20%), or both (20%). In the disease-free group (n=9), most patients presented non-functioning tumors (66.7%). The median initial tumor size was 105 mm, the median Ki67 index was 20%, and the median Weiss score was 7. No evidence of disease recurrence was observed during a median follow-up period of 55 months of regular monitoring.

**Table 2:** Clinical characteristics of subgroups of ACC patients stratified by disease status (Preoperative, Recurrent and Disease-free ACC patients).

Characteristics	Preoperative ACC (n=6)	Recurrent ACC (n=10)	Disease-free ACC (n=9)	p-value (pairwise comparisons)
Age at diagnosis, years median (range)	66.5 (33-76)	49 (37-89)	55 (41-72)	ns
Sex: M, n (%)	2 (33.3%)	4 (40%)	4 (44.4%)	ns
Initial hormonal status, n (%)	1 (16.7%) nf, 5 (83.4%) C (all with CS)	1 (10%) nf, 1 (10%) C (CS), 8 (80%) A and C	6 (66.7%) nf, 3 (33.3%) C (all with CS)	0.003 (preoperative *vs* recurrent), 0.047 (preoperative *vs* disease-free), 0.001 (recurrent *vs* disease-free)
Primary tumor size, mm median (range)	99.5 (57-120)	110 (52-185) †	105 (58-120)	ns
Initial stage, n (%)	III (n=3, 50%), IV (n=3, 50%)	II (n=2, 20%), III (n=6, 60%), IV (n=2, 20%)	II (n=5, 55.6%), III (n=4, 44.4%)	ns
Resection status, n (%)	R0 (n=2, 33, 3%), R1 (n=2, 33, 3%), R2 (n=2, 33, 3%)	R0 (n=7, 70%), R1 (n=1, 10%), R2 (n=2, 20%) †	R0 (n=9, 100%)	ns
Initial Ki67%, median (range)	30 (5-80)	39 (20-50) †	20 (5-50)	ns
Initial Weiss score, median (range)	6.5 (4-7)	6 (6-7) †	7 (4-9)	ns
Tumor presence at sample taking, n (%)	Primary tumor (3,50%), Primary tumor and distant metastases (3, 50%)	Local recurrence (4, 40%), Distant metastases (in lung 2, 20%, in liver 2, 20%, both in lung and liver 2, 20%)	No evidence of disease (9, 100%)	na
Adjuvant treatment received till sample taken, n (%)	None, 6 (100%)	MTT* (n=4, 40%), MTT+LT* (n=2, 20%), MTT+LT+K* (n=2, 20%)	MTT*(n=5, 55.6%), MTT+LT* (n=2, 22.9%)	na
Time to recurrence, months median (range)	6**	4 (3-14)	na	ns
1-year survival rate (%)	83.3%***	100%	100%	ns
Follow-up period, months median (range)	12 (12-24)	12(12-60)	55 (32-183)	0.001 and 0.003 for the comparison of active and recurrent ACC with the disease-free state, respectively

ACC, adrenocortical carcinoma; na, not applicable; ns, non-significant; nf, non-functioning; C, cortisol-producing; A, androgen-producing; MTT, mitotane alone; MTT+LT, mitotane combined with local therapy; MTT+LT+K, mitotane combined with local therapy and chemotherapy.

*The median time to reach mitotane plasma levels within the therapeutic range (>14 mg/L) was 14 weeks in patients with recurrent disease and 12 weeks in disease-free patients.

** During the follow-up period, only one patient presented with recurrence.

*** One patient with cortisol-secreting ACC experienced a fatal outcome due to acute pulmonary edema 5 months after diagnosis.

† The median tumor size of recurrent tumors was 35 (30-65) mm, calculated using the maximum lesion size for each patient with multiple lesions. Recurrence was surgically treated in 3 patients (R1, n=2; R0, n=1), with histopathological analysis showing a median Ki-67 index of 30% (10%-40%) and a Weiss score of 6 (6-7).

ANOVA, Kruskal-Wallis, chi-square, or Fisher’s exact tests were applied to assess differences between groups as appropriate, followed by *post-hoc* analyses for pairwise comparisons.

The preoperative samples were categorized into three groups based on functionality: non-functioning (n=26, including 1 ACC and 25 ACA patients), MACS (n=22, all ACA), and CS (n=8, including 5 ACC and 3 ACA patients) (**Table 3**). Post-ODST cortisol levels were significantly higher in both the CS and MACS groups compared to the non-functioning group (p<0.001 for both comparisons). ACTH levels were suppressed in the CS group compared to non-functioning (p=0.002), with no significant differences between MACS and either the non-functioning or CS group. Additionally, UFC levels were elevated in the CS group compared to non-functioning (p=0.03), but no differences were found between MACS and either the non-functioning or CS group. Hypertension was more common in the MACS (59.1%) and CS (75%) groups than in the non-functioning group (30.8% (p=0.05 and p=0.03, respectively), whereas no significant differences were observed for other metabolic complications.

**Table 3:** Baseline characteristics of AI patients evaluated preoperatively.

Characteristics	NFAT (n=26)	MACS (n=22)	CS (n=8)	p-value (NFAI *vs* MACS, NFAI *vs* CS, MACS *vs* CS)
Age, years (mean ± SD)	61.1 ± 10.8	65.9 ± 9.7	57.8 ± 12.6	ns
Sex: M, n (%)	3 (11.5%)	11 (50%)	4 (50%)	0.05, ns, ns
Size, mm (mean ± SD)	24.03 ± 14.5	32.2 ± 13.9	82.3 ± 28.6	ns, 0.001, ns
BMI (mean ± SD)	27.1 ± 6.2	26.4 ± 4.6	27.8 ± 4	ns
Hypertension, n (%)	8 (30.8%)	13 (59.1%)	6 (75%)	0.05, 0.03, ns
Dyslipidemia, n (%)	18 (69.2%)	16 (72.7%)	5 (62.5%)	ns
Prediabetes/DM2, n (%)	11 (42.3%)	13 (59.1%)	5 (62.5%)	ns
Osteopenia/osteoporosis, n (%)	9/14 (64%) *	9/15 (60%) *	2/2 (10%) *	ns
F post-ODST, μg/dL (mean ± SD)	1.07 ± 0.32	3.03 ± 0.86	20.6 ± 9.2	<0.001, <0.001, ns
ACTH, pg/mL (mean ± SD)	30.39 ± 22.7	17.24 ± 9.8	7.9 ± 9.7	ns, 0.002, ns
UFC, μg/24h(mean ± SD)	99.4 ± 68.4	103.4 ± 108.6	418.5 ± 336.1	ns, 0.03, ns

M, male; BMI, body mass index; DM2, diabetes mellitus type 2; SD, standard deviation; ODST, overnight dexamethasone suppression test; F, serum free cortisol; UFC, 24-hour urinary free cortisol; ACTH, adrenocorticotropic hormone; NFAT, non-functioning adrenocortical tumors; MACS, mild autonomous cortisol secretion; CS, Cushing syndrome; ns, non significant.

*Data regarding osteopenia/osteoporosis in patients with NFATs, MACS and CS were available in 14, 15 and 2 patients, respectively.

ANOVA, Kruskal-Wallis, chi-square, or Fisher’s exact tests were applied to assess differences between groups as appropriate, followed by *post-hoc* analyses for pairwise comparisons.

### 3.2 Analysis of circulating miRNA expression in patients with ATs

When the ACC cohort was analyzed as a single, unstratified group, miR-483-5p and miR-210 levels were elevated compared to the ACA group, with p-values of 0.02 and 0.03, respectively, while no statistically significant difference was observed compared with the control group (p-values = 0.2 and 0.6, respectively). No significant difference was observed in the levels of miR-335 between the groups. Furthermore, ROC analysis demonstrated a modest discriminatory ability of these biomarkers. Specifically, the AUC for miR-483-5p in distinguishing ACC from ACA was 0.7 (p = 0.01), while the AUC for miR-210 was 0.69 (p = 0.01). The ROC analysis did not reveal a statistically significant discriminatory ability for either biomarker in differentiating ACC from control samples.

**Figure 1:**
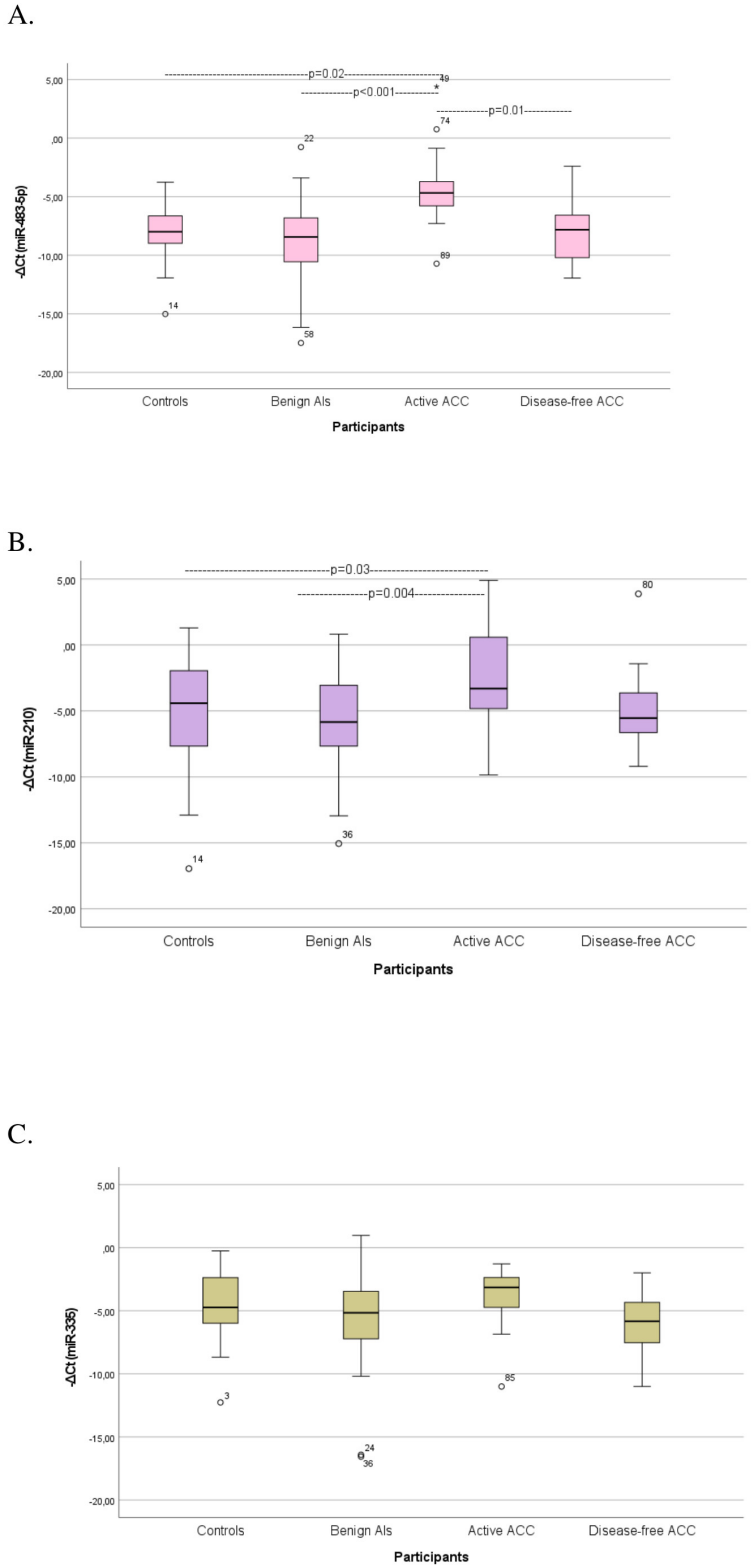
Comparison of serum miRNA levels **(A)** miR-483-5p, **(B)** miR-210, **(C)** miR-335) among controls, patients with benign Ats (ACAs), patients with active ACC, and disease-free ACC cases. Kruskal-Wallis test was applied to assess differences between the groups, followed by *post-hoc* analysis for pairwise comparisons. ATs, Adrenocortical tumors; ACA, Adrenocortical adenoma; ACC, Adrenocortical carcinoma.

Stratification of the ACC cohort by the presence of tumor burden resulted in an active group, comprising a diverse population of preoperative and recurrent patients, and a disease-free group, including treated ACC patients considered without relapse during the follow-up. Circulating miR-483-5p was significantly elevated in patients with active ACC compared with patients with ACAs (p<0.001) and controls (p=0.02) (**Supplementary Table 1A**) (**Figure 1A**). MiR-483-5p was also considerably elevated in patients with active ACC compared with disease-free ACC patients (p=0.01) (**Supplementary Table 1A**) (**Figure 1A**). Circulating miR-483-5p levels presented a 24-fold, 14-fold and 17-fold overexpression in patients with active ACC compared with those with ACAs, controls and disease-free ACC patients, respectively. Circulating miR-210 levels were significantly elevated in active ACC patients compared with patients with αCA (p=0.004) and controls (p=0.03) (**Supplementary Table 1A**) (**Figure 1B**), showing a 9-fold and 8-fold over-expression in active ACC patients compared with the 2 other groups, respectively. Circulating miR-335 levels showed no statistically significant difference in their expression among the 4 studied groups (**Supplementary Table 1A**) (**Figure 1C**).

**Figure 2:**
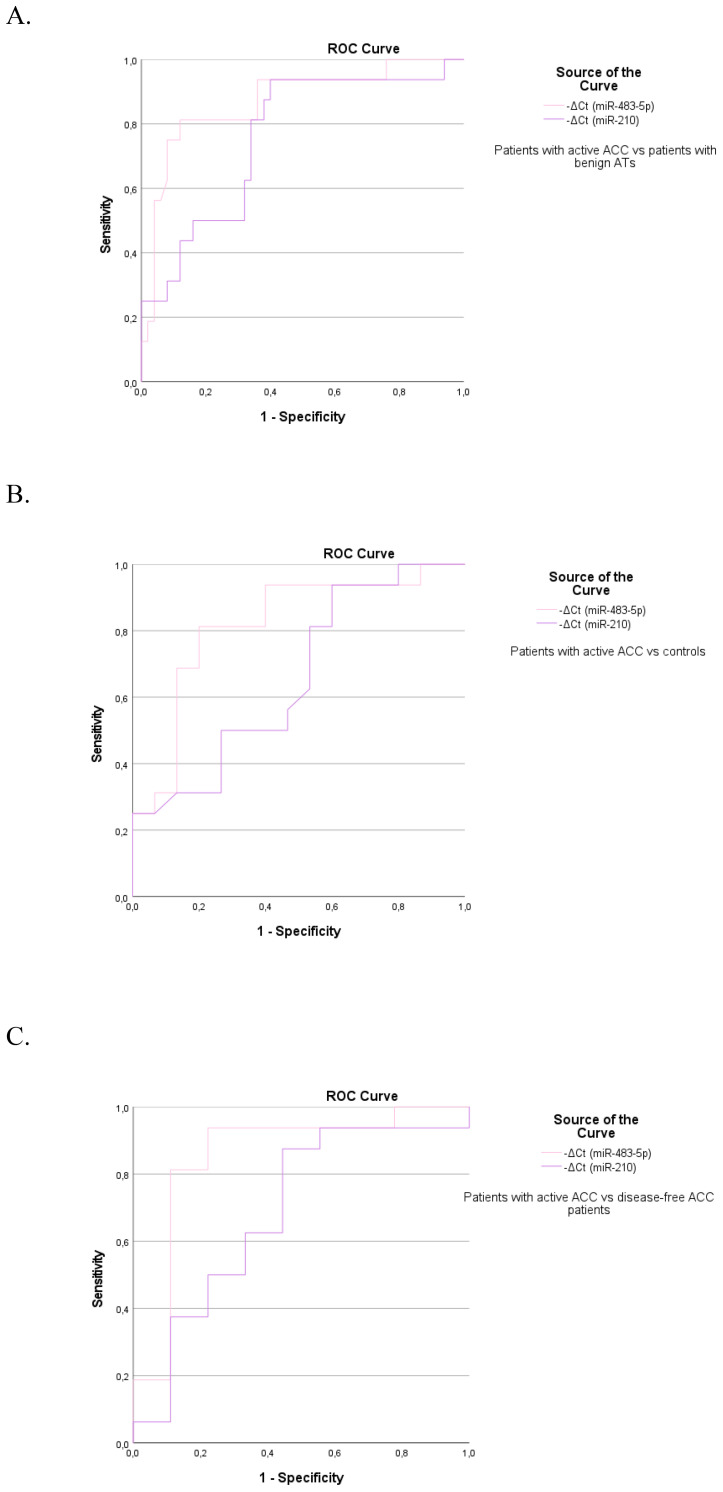
The ROC curve indicates the diagnostic accuracy of circulating miR-483-5p and miR-210 in discriminating **(A)** patients with active ACC from those with benign ATs (0.869, 95%CI: 0.761–0.978, p<0.001 and 0.759, 95%CI: 0.623-0.894, p=0.002 respectively), **(B)** patients with active ACC from controls (0.817, 95%CI: 0.659–0.974, p=0.003 and 0.658, 95%CI: 0.464-0.853, p=0.13 respectively), **(C)** patients with active ACC from disease-free ACC patients (0.854, 95%CI: 0.672–1, p=0.004 and NS respectively).

High diagnostic accuracy for discriminating patients with active ACC from those with ACAs was observed for circulating miR-483-5p with an AUC of 0.869 (95%CI: 0.761–0.978, p<0.001). MiR-210 achieved sufficient diagnostic accuracy with an AUC of 0.759 (95%CI: 0.623-0.894, p=0.002) (**Supplementary Table 2**) (**Figure 2A** **).** The best diagnostic performance in terms of high sensitivity (81.3%) and specificity (88%) was noted with miR-483-5p. Serum miR-483-5p levels showed also high diagnostic accuracy in differentiating patients with active ACC from controls (AUC=0.817, 95%CI: 0.659–0.974, p=0.003)(**Figure 2B**) and disease-free ACC patients (AUC=0.854, 95%CI: 0.672–1, p=0.004) (**Figure 2C**), reaching sensitivity of 81.3% and specificity of 80% in the first case, and 81.3% sensitivity and 89% specificity in the second, respectively (**Supplementary Table 2**).

**Figure 3:**
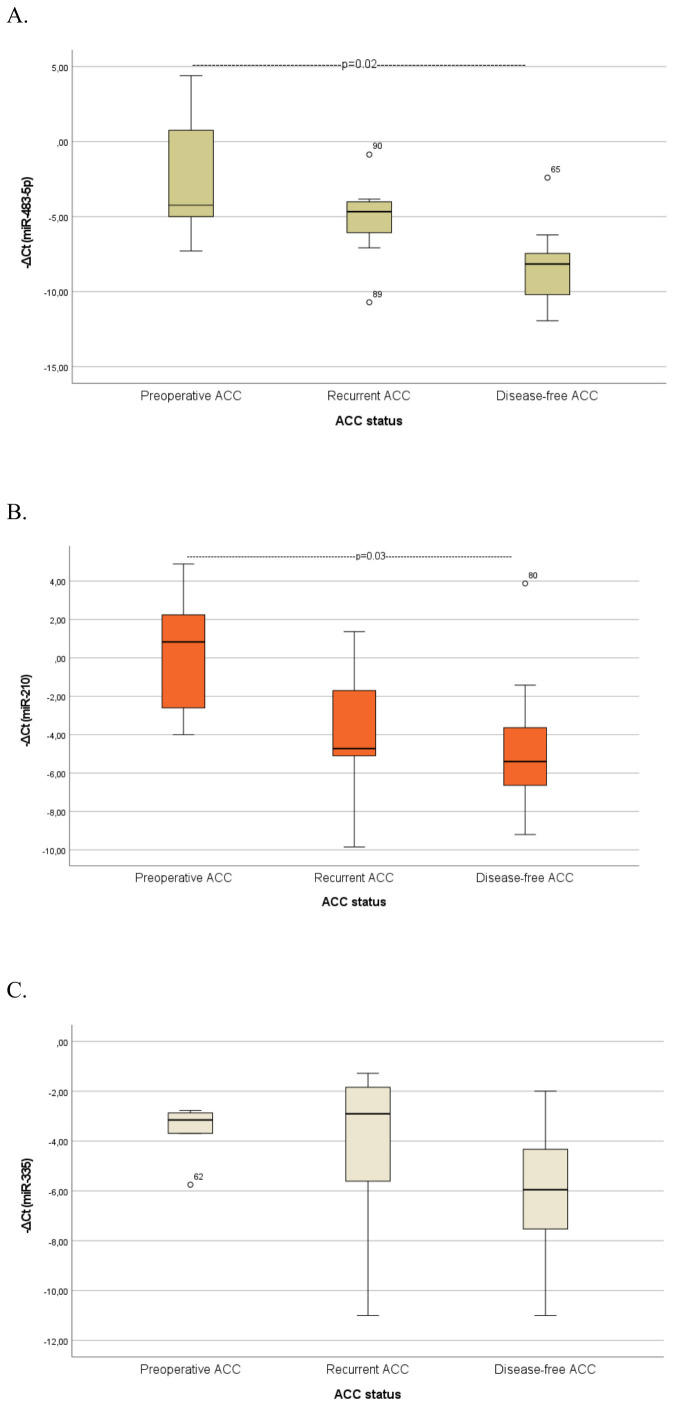
Comparison of serum miRNA levels **(A)** miR-483-5p, **(B)** miR-210, **(C)** miR-335) between patients with preoperative ACC, patients with recurrent ACC and disease-free ACC patients. Kruskal-Wallis test was applied to assess differences between the groups, followed by *post-hoc* analysis for pairwise comparisons. ATs, Adrenocortical tumors; ACA, Adrenocortical adenoma; ACC, Adrenocortical carcinoma.

A further analysis was conducted to evaluate serum biomarker levels across ACC patients with different disease status (**Figure 3**), (**Supplementary Table 1B**). Serum miR-483-5p levels were consistently elevated in both preoperative (p=0.02) and recurrent ACC cases compared with disease-free cases. However, the comparison between recurrent and disease-free cases approached, but did not reach statistical significance (p = 0.06). MiR-210 levels were significantly higher in preoperative ACC cases compared to disease-free cases (p = 0.03). Both biomarkers exhibited a trend toward higher levels in preoperative cases compared to recurrent cases, however, the observed differences were not statistically significant.

**Figure 4:**
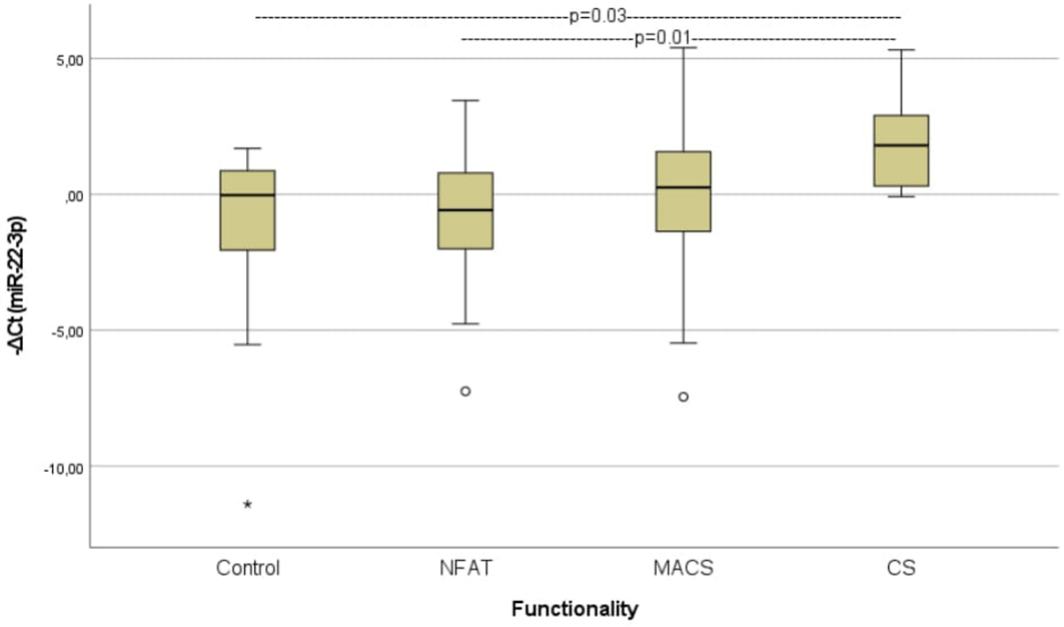
Comparison of serum miR-22-3p levels among controls, patients with non-functioning ATs, MACS and CS. Kruskal-Wallis test was applied to assess differences between the groups, followed by *post-hoc* analysis for pairwise comparisons. NFAT, Non-functioning adrenal adrenocortical tumors; MACS, mild autonomous cortisol secretion; CS, Cushing syndrome.

**Figure 5:**
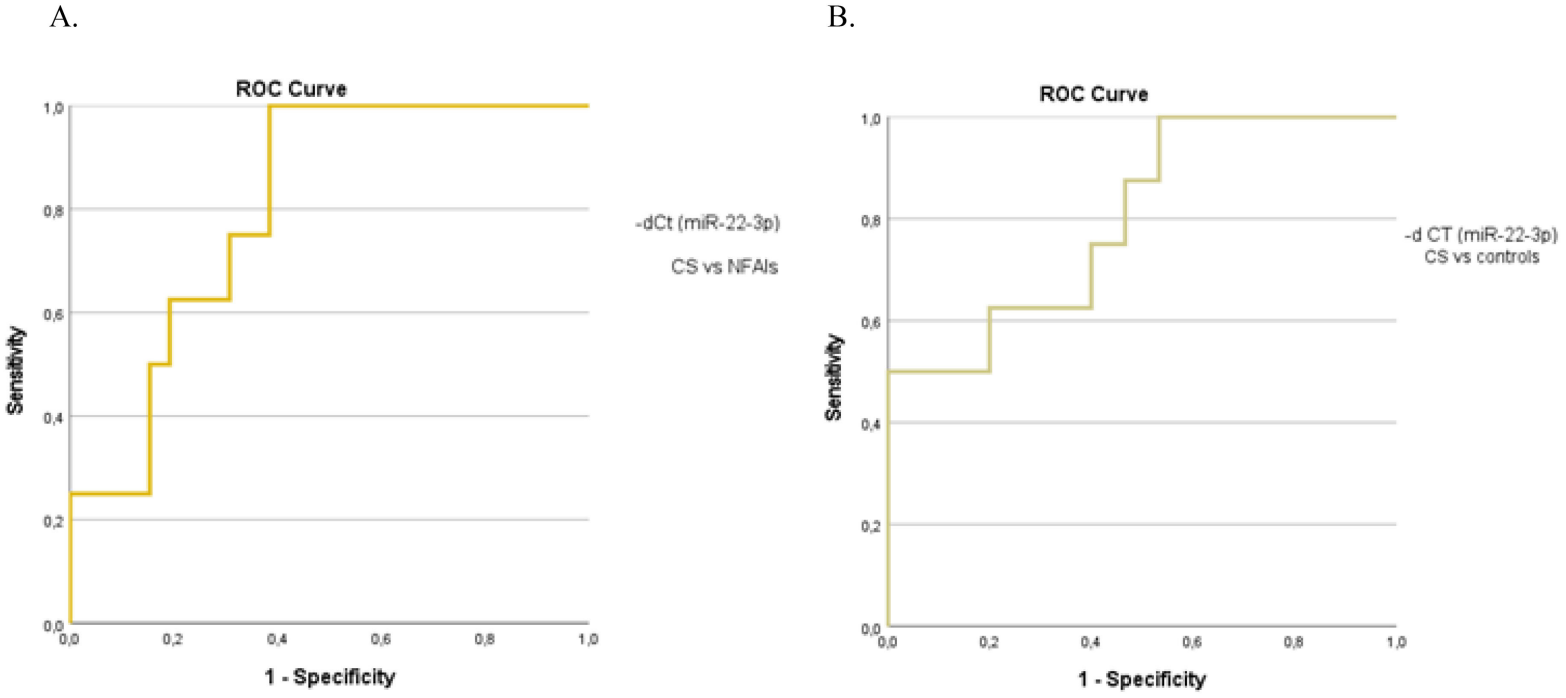
The ROC curve indicates the diagnostic performance of circulating miR-22-3p in discriminating **(A)** patients with CS from those with non-functioning ATs (0.800, 95%CI: 0.653–0.953, p=0.01) **(B)** patients with CS from controls (0.800, 95%CI: 0.610–0.990, p=0.02).

When analyzing the preoperative groups based on functionality, circulating miR-22-3p levels were significantly elevated in patients with CS compared with those with non-functioning ATs (p=0.01) and controls (p=0.03) (**Figure 4**). The molecular marker showed high diagnostic accuracy in discriminating patients with CS from those with non-functioning ATs (AUC=0.800, 95%CI: 0.653–0.953, p=0.01) and controls (AUC= 0.800, 95%CI: 0.610–0.990, p=0.02) (**Figure 5**). However, it was not sufficient to differentiate patients with MACS from those with CS or from non-functioning ATs (p=0.08 and p=0.35, respectively).

**Figure 6:**
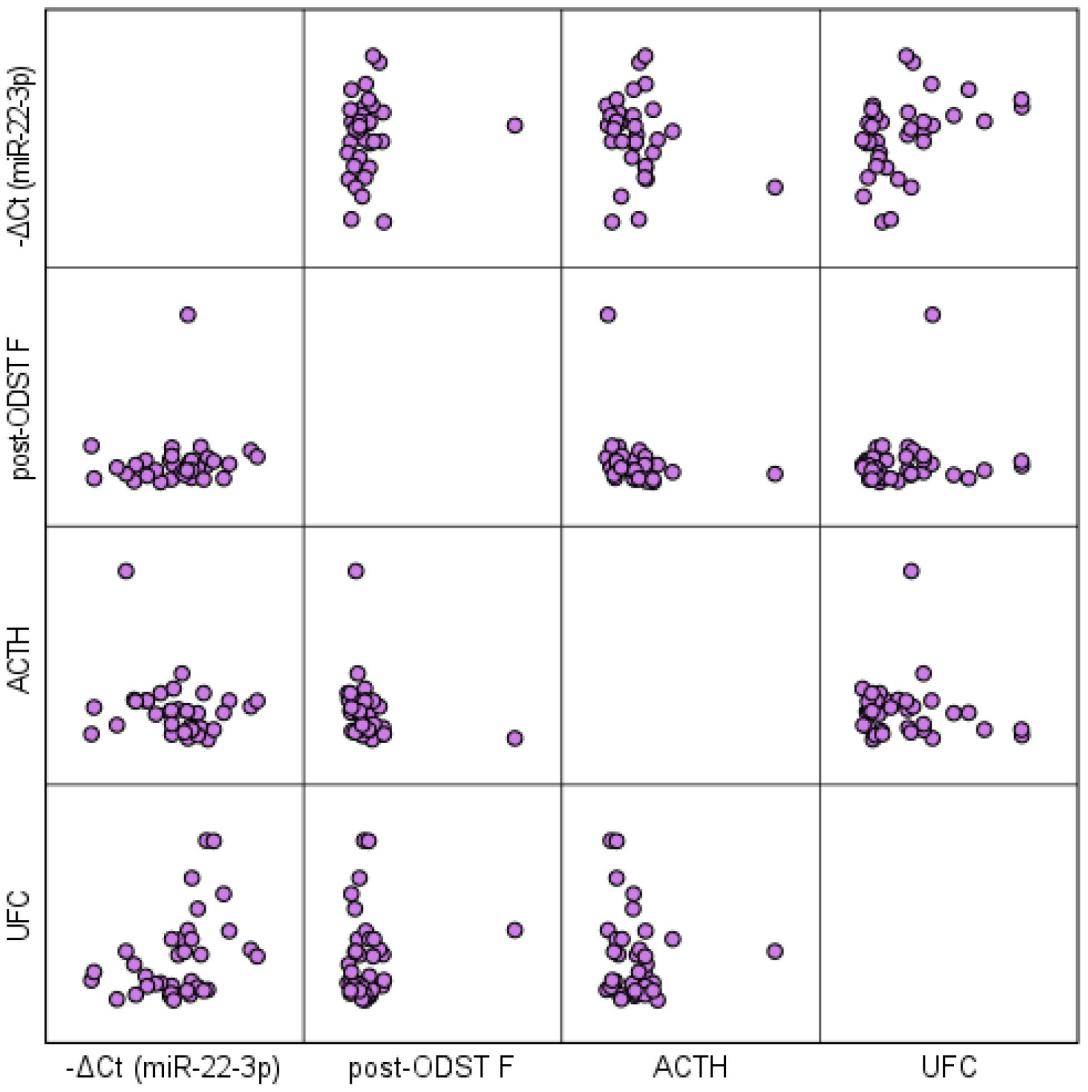
Scatterplot matrix illustrating the correlation between circulating miR-22-3p levels and traditional diagnostic tests for hypercortisolism in patients with ATs. ODST, overnight dexamethasone suppression test; F, serum free cortisol; UFC, 24-h urinary free cortisol; ACTH, adrenocorticotropic hormone.

Correlation analysis between circulating miR-22-3p levels and the traditional diagnostic tests for hypercortisolism reveals a positive correlation with serum cortisol levels post-ODST (rs=0.361, p=0.005) and UFC (rs=0.476, p<0.001), and a negative correlation with ACTH (rs=-0.351, p=0.006) in patients with ATs (**Figure 6**). No significant association was observed between circulating miR-22-3p levels and tumor size. The biomarker demonstrated limited predictive value for the presence of metabolic complications, including hypertension, obesity, dyslipidemia, osteopenia/osteoporosis, and type 2 diabetes mellitus, as indicated by insufficient sensitivity and specificity in the ROC analysis.

## 4 Discussion

The differential diagnosis of ATs often involves a multi-step process of thorough laboratory testing and imaging studies, which nevertheless may remain inconclusive. Our study highlights the potential of circulating miR-483-5p and miR-210 as promising non-invasive biomarkers for distinguishing active ACC cases from ACA. Notably, the absence of miR-483-5p expression in disease-free ACC patients, where tumor burden is low or absent, suggests that this biomarker may not only be useful for diagnostic purposes but also hold promise for disease monitoring. Although the exact mechanism remains to be elucidated, our findings support the consideration of miR-483-5p and miR-210 as tumor-derived biomarkers. Additionally, we validated the effectiveness of miR-22-3p in detecting overt hypercortisolism, although the marker was not reliable in distinguishing patients with MACS.

Given the rarity of ACC cases, data on potential non-invasive biomarkers are limited. Small-cohort studies have explored the differential expression of miRNAs in various body fluids (plasma, serum, urine) among patients with ACCs and ACAs, following their initial identification in tissue samples. In 7 studies using blood samples, miR-483-5p was consistently found to be overexpressed in patients with active ACC compared to ACA or control samples (6–8, 10–13), though no significant differences were observed when tested in urinary samples (13). In a study of 26 ACC patients, circulating miR-483-5p levels, measured within three months post-surgery, were found to be prognostic for both recurrence and overall survival (16). Our study further identified a significant decrease in miR-483-5p levels in disease-free ACC patients during long-term follow-up, suggesting the potential of miR-483-5p as a biomarker for monitoring disease progression.

Although several studies identified the differential tissue expression of miR-210 and miR-335 between ACC and ACA (5), only one study for each tested their potential as liquid biopsy components (6, 10).Circulating miR-210 was overexpressed in patients with active ACC in a study involving 4 ACC patients (10), while miR-335 was downregulated in a study including 23 ACC patients (6), although this latter finding was not confirmed in our study. Furthermore, the potential of circulating miRNAs, specifically miR-483-5p and miR-210, to monitor the efficacy of ACC treatment has been limitedly explored in mice xenografted with ACC cell lines. Preliminary preclinical findings have provided evidence that tumor reduction observed after therapy may be reflected in miRNA levels, indicating that these miRNAs could serve as potential markers of treatment response (17, 18). Specifically, circulating miR-483-5p levels were significantly suppressed in an ACC xenograft mouse model responding to treatment with a combination of 9-cis retinoic acid and mitotane (17), whereas in a study involving the SW-13 tumor model, circulating miR-210 levels were found to be elevated following treatment with LEDP-M (etoposide, liposomal doxorubicin, liposomal cisplatin, and mitotane) (18). In our study, miR-483-5p levels were found to be significantly lower in patients who had responded to prior therapy and showed no evidence of residual disease, compared to those with active ACC. Early investigations into other components of liquid biopsy, such as circulating tumor cells and circulating tumor DNA, have shown promising results in distinguishing between malignant and benign ATs (3, 19).

Limited data are, also, available regarding the role of miRNAs in differentiating cortisol-secreting from non-functioning ATs, although the need for novel biomarkers to identify patients with clinically significant cortisol excess is well-established (1). Expanding on the findings of a preliminary study on tissue samples (20), a single study evaluated the diagnostic potential of selected miRNAs in serum samples from patients with non-functioning ATs and those with overt hypercortisolism (9). In this study, circulating miR-22-3p emerged as the most effective biomarker, displaying significant overexpression in individuals with CS (either with malignant or benign tumors), which is consistent with our findings. Additionally, miR-22-3p levels exhibited a significant positive correlation with urinary-free cortisol (rs=0.76, p<0.0001) and cortisol levels following a low-dose dexamethasone suppression test (rs=0.56, p=0.02).

Although the deregulation of the selected miRNAs implies their involvement in the pathogenesis of ATs, the precise mechanisms remain unclear. MiR-483 gene locus has been mapped within intron 2 of insulin-like growth factor 2 (IGF2) gene, which is frequently overexpressed in ACC cases (21). Consequently, it has been hypothesized that these two genes are co-expressed. However, miR-483-5p appeared, also, to have a functional role promoting migration and invasiveness as well as cell proliferation in ACC cell models (22, 23). Experimental data across various cancer types suggest that miR-210 is involved in the hypoxia pathway. In human ACC cases, elevated miR-210 tissue expression has been linked to hypoxia-related factors, including necrosis and the expression of glucose transporter 1 (GLUT-1) (24). Potential target genes for miR-335 have been proposed through computational analysis, but experimental validation has not been performed to confirm the actual interactions (25). In the case of miR-22-3p, in silico prediction revealed the presence of a glucocorticoid response element (GRE) within the gene promoter, explaining its overexpression in cortisol-producing tumors (9).

Although micro-RNAs possess features that qualify them as potential biomarkers, including stability in circulation, tissue specificity, and high accuracy in quantification using current detection methods, their application in clinical practice remains limited due to a lack of validating studies and their inherent heterogeneity (26). Interindividual variability in miRNA levels has been observed, as evidence suggests that epidemiological and lifestyle factors can influence their levels. Additionally, the absence of standardized methods further complicates their consistency and reliability in clinical settings.

Although our study provides preliminary insights into the use of liquid biopsy in patients with ATs, we acknowledge certain limitations. The primary limitation is the inclusion of a heterogeneous population of ACC patients within the ‘active disease’ category, comprising individuals with primary tumors as well as those experiencing local recurrence or distant metastases following treatment of the primary tumor. Variations in disease status and prior treatment history may have influenced the circulating biomarker expression, which must be taken into consideration when interpreting the findings. Nevertheless, given the rarity and aggressive nature of ACC, assembling a large and uniform cohort poses a significant challenge. A promising direction for future research would be the examination of serial serum samples from ACC patients across various stages of the disease—preoperatively, postoperatively, during recurrence, in response to treatment, and after remission- to uncover dynamic changes in miRNA expression throughout disease progression and in response to treatment. Unfortunately, our study did not include the necessary longitudinal samples to test this hypothesis. Furthermore, we did not assess the expression of miRNAs in the corresponding tumor tissues, which would be essential to confirm the origin of the circulating miRNAs. It is essential to emphasize that a large study involving groups of patients with various types of ATs would be valuable for evaluating the specificity of these molecular markers, given the evidence of overlap among different tumor types (26).

## 5 Conclusion

Our study demonstrates the promising potential of circulating miRNAs as supplementary tools for the diagnosis and monitoring of ATs, suggesting a non-invasive, precision-based approach to complement traditional diagnostic methods. We highlight the diagnostic potential of miR-483-5p and miR-210 in differentiating malignant from benign ATs, as well as their additional value in distinguishing active disease from disease-free cases. Additionally, miR-22-3p shows promise in identifying overt hypercortisolism, though it is not effective for distinguishing MACS. Further validation in large prospective cohorts is essential to standardize miRNA analysis and integrate these biomarkers into clinical settings. A large-scale, multi-center study incorporating longitudinal data would be invaluable in refining our understanding of miRNA dynamics and their potential role in diagnosing and monitoring ATs, with further applications in the therapeutic field.

## Data Availability

The raw data supporting the conclusions of this article will be made available by the authors, without undue reservation.
