# Supplementary material for: Evaluation of microRNAs as liquid biopsy markers in adrenocortical tumors
Source: Front Endocrinol (Lausanne). 2025 Jan 30;16:1511520. doi: 10.3389/fendo.2025.1511520 (PMC11821491; doi:10.3389/fendo.2025.1511520)
Supplement: Supplementary file 1 [file Table1.docx]

**Supplementary Table 1**

**A. Comparative analysis of circulating miRNA levels among patient groups: Active ACC** (n=16)**, ACA** (n=50)**, disease-free ACC** (n=9)**, and controls** (n=15)**.**

| **Comparison** | **miR-483-5p (p-value)** | **miR-210 (p-value)** | **miR-335 (p-value)** |
| --- | --- | --- | --- |
| **Active ACC vs ACA** | Elevated (p<0.001) | Elevated (p=0.004) | Comparable (p=0.1) |
| **Active ACC vs controls** | Elevated (p=0.02) | Elevated (p=0.03) | Comparable (p=0.9) |
| **Active ACC vs disease-free ACC** | Elevated (p=0.01) | Comparable (p=0.8) | Comparable (p=0.1) |
| **ACA (n=50) vs controls (n=15)** | Comparable (p=0.9) | Comparable (p=0.9) | Comparable (p=0.9) |
| **ACA vs disease-free ACC** | Comparable (p=0.9) | Comparable (p=0.9) | Comparable (p=0.9) |
| **Controls vs disease-free ACC** | Comparable (p=0.9) | Comparable (p=0.9) | Comparable (p=0.8) |

**B. Comparative analysis of circulating miRNA levels among ACC patients in different disease status: Preoperative** (n=6), **Recurrent** (n=10), **Disease-free** (n=9) **ACC patients.**

| **Comparison** | **miR-483-5p (p-value)** | **miR-210 (p-value)** | **miR-335 (p-value)** |
| --- | --- | --- | --- |
| **Preoperative ACC vs Recurrent ACC** | Comparable (p=0.9) | Comparable (p=0.9) | Comparable (p=0.9) |
| **Preoperative ACC vs Disease-free ACC** | Elevated (p=0.018) | Elevated (p=0.02) | Comparable (p=0.23) |
| **Recurrent ACC vs Disease-free ACC** | Comparable (p=0.06) | Comparable (p=0.07) | Comparable(p=0.07) |

Abbreviations: ACC: adrenocortical carcinoma, ACA: adrenocortical adenoma

**ANOVA or Kruskal-Wallis tests were applied to assess differences between groups, , depending on data normality, followed by post-hoc analysis for pairwise comparisons.
