# Supplementary material for: Evaluation of microRNAs as liquid biopsy markers in adrenocortical tumors
Source: Front Endocrinol (Lausanne). 2025 Jan 30;16:1511520. doi: 10.3389/fendo.2025.1511520 (PMC11821491; doi:10.3389/fendo.2025.1511520)
Supplement: Supplementary file 2 [file Table2.docx]

**Supplementary Table 2. Diagnostic performance of circulating miR-483-5p and miR-210 in discriminating patients with active ACC (n=16) from patients with ACA (n=50), disease-free ACC patients (n=9) and controls (n=15).**

| **MiRNA** | **Comparison** | **AUC** | **95% CI** | **p-value** | **Sensitivity** | **Specificity** |
| --- | --- | --- | --- | --- | --- | --- |
| **MiR-483-5p** | Active ACC vs ACA | 0.869 | 0.761-0.978 | p<0.001 | 81.3% | 88% |
|  | Active ACC vs controls | 0.817 | 0.659-0.974 | p=0.003 | 81.3% | 80% |
|  | Active ACC vs disease-free ACC | 0.854 | 0.672-1 | p=0.004 | 81.3% | 89% |
| **MiR-210** | Active ACC vs ACA | 0.759 | 0.623-0.894 | P=0.002 | 87.5% | 62% |

Abbreviations; ACC: adrenocortical carcinoma, ACA: adrenocortical adenoma, AUC= area under the curve, CI: confidence intervals.

* AUC p-values were calculated using DeLong’s test to assess the statistical significance. Only statistically significant results shown in the table.
